# Questionable prospective effects on burnout and exhaustion: simulated reanalyses of cross-lagged panel models

**DOI:** 10.3389/fpsyg.2025.1618120

**Published:** 2025-08-29

**Authors:** Kimmo Sorjonen, Bo Melin, Filippa Folke, Marika Melin

**Affiliations:** Karolinska Institutet (KI), Solna, Sweden

**Keywords:** burnout and exhaustion, causality, complementary models, cross-lagged panel model, scrutiny, simulated data, artifactual findings

## Abstract

Burnout and exhaustion has been extensively studied in organizational, work, and health psychology. Studies using the cross-lagged panel models have tended to conclude, explicitly or implicitly (e.g., in the form of policy recommendations), causal prospective effects of, for example, organizational demands, job insecurity, and depression on burnout and exhaustion. However, it is well established that effects in the cross-lagged panel model may be artifactual, e.g., due to correlations with residuals and regression to the mean. Here, we scrutinized 23 previously reported prospective effects on burnout/exhaustion by fitting complementary models to data that were simulated to resemble data in the evaluated studies. With one possible exception, the previously reported prospective effects did not withstand scrutiny, i.e., they appeared to be artifactual. It is important for researchers to bear in mind that correlations, including effects in cross-lagged panel models, do not prove causality in order not to overinterpret findings. We recommend researchers to scrutinize findings from cross-lagged panel models by fitting complementary models to their data. If findings from complementary models converge, conclusions are corroborated. If, on the other hand, findings diverge, caution is advised and claims of causality, explicit or implicit, should probably be avoided.

## Introduction

Burnout, or, more specifically, job/occupational burnout, is a condition characterized by “emotional exhaustion, depersonalization, and reduced personal accomplishment that can occur among individuals who work with people in some capacity” (Maslach et al., 1996, p. 192). In agreement with this characterization, burnout is often assumed to be defined, and assessed, by three dimensions: exhaustion, cynicism/depersonalization, and inefficiency (Maslach et al., 2001; Maslach and Leiter, 2016). Burnout and related constructs, e.g., exhaustion, have received a lot of attention in organizational, work, and health psychology. Here, we do not wish to take a stance on exactly what burnout is and if exhaustion is the same, related to, or something completely different compared with burnout. We have followed the vocabulary in the original papers (more on this below). What authors of the original papers claim to have predicted, and what measuring instruments they have used, is included in our descriptive dataset available at the Open Science Framework at https://osf.io/smy5n/.

Studies have reported correlations between burnout/exhaustion and, for example, workload, supervisor support, turnover intentions, and organizational commitment (Lee and Ashforth, 1996). Among nurses, burnout has also been found to correlate positively with the number of employment relationships (Alves et al., 2023) and with sleep problems (Lin et al., 2024). However, correlations do not prove that burnout causally affects or is affected by turnover intentions, workload, etc., as the correlations may be due to effects by confounding variables (Reichenbach, 1971).

In the cross-lagged panel model (CLPM), a subsequent measure of some outcome variable Y is regressed on a prior measure of a predictor X as well as a prior measure of the outcome Y, and vice versa. The cross-lagged effect of prior X on subsequent Y while adjusting for prior Y is often assumed to allow stronger causal inference than zero-order correlations and cross-lagged effects are often described using explicit or implicit (e.g., in the form of policy recommendations) causal language. For example, based on results from analyses with CLPM, Tóth-Király et al. (2021) concluded that burnout and depression mutually reinforce each other.

However, it is well established that adjusted cross-lagged effects may be artifactual, e.g., due to correlations with residuals and regression toward the mean (Campbell and Kenny, 1999; Glymour et al., 2005; Eriksson and Häggström, 2014; Castro-Schilo and Grimm, 2018; Sorjonen et al., 2019; Lucas, 2023). For example, due to a positive correlation between depression and burnout, we should expect a higher true degree of burnout, and consequently a more negative residual in the initial measurement of burnout, among individuals with higher measured depression compared with individuals with the same initial measured burnout but with lower measured depression. However, as residuals tend to regress toward a mean value of zero between measurements, we should expect a more positive, but artifactual, change in measured degree of burnout among those with higher measured degree of depression compared with those with the same initial measured degree of burnout but with lower measured degree of depression. This combination of correlations with residuals and regression toward the mean might explain the positive effect of initial depression on subsequent burnout when adjusting for initial burnout reported by Tóth-Király et al. (2021).

We have previously reported results suggesting that many conclusions of prospective effects on work engagement, e.g., by job control and depressive symptoms, may have been based on artifactual findings and, consequently, inaccurate (Sorjonen et al., 2024b). The objective of the present study was to conduct a similar analysis of reported prospective effects on burnout and exhaustion in studies using CLPM and to evaluate if the effects may have been artifactual rather than genuine.

## Method

We identified 13 studies using CLPM and claiming prospective effects on either burnout or exhaustion. Some studies presented more than one cross-lagged effect, either due to including more than one predictor of burnout/exhaustion or due to analyzing data from more than two waves of measurement. Consequently, we reanalyzed a total of 23 cross-lagged effects. We refer to the reanalyzed studies for more comprehensive information on procedures, samples, etc. Some key components are presented in Table 1 and more information is included in our descriptive dataset available at the Open Science Framework at https://osf.io/smy5n/.

**Table 1:** Characteristics of the simulated and reanalyzed studies.

S. E.	Study	*N*	Male	Age	Wave	Predictor	*b*
1.1	Ângelo and Chambel (2015) ^e^	651	90%	35	1 to 2	Organizational demands	0.11
2.1	De Cuyper et al. (2012) ^e^	1,314	32%	43	1 to 2	Job insecurity	0.05
3.1	Houkes et al. (2008) ^e^	261	52%	46	1 to 2	Work family interference	0.14
3.2	Houkes et al. (2008) ^e^	261	52%	46	1 to 2	Avoidance	0.10
3.3	Houkes et al. (2008) ^e^	261	52%	46	1 to 2	Perfectionism	0.12
4.1	Innstrand et al. (2008) ^e^	2,235	54%	45	1 to 2	Work to family conflict	0.34
4.2	Innstrand et al. (2008) ^e^	2,235	54%	45	1 to 2	Work to family facilitation	−0.24
4.3	Innstrand et al. (2008) ^e^	2,235	54%	45	1 to 2	Family to work conflict	0.06
5.1	Jensen (2016) ^e^	1703	78%	-	1 to 2	Work role conflict	0.06
5.2	Jensen (2016) ^e^	1703	78%	-	1 to 2	Work family conflict	0.06
6.1	Jensen and Knudsen (2017) ^e^	1702	78%	-	1 to 2	Psychological health complaints	0.16
7.1	Kinnunen et al. (2019) ^e^	664	42%	48	1 to 2	Affective rumination	0.14
7.2	Kinnunen et al. (2019) ^e^	664	42%	48	2 to 3	Affective rumination	0.11
8.1	Liu et al. (2021) ^b^	1,226	50%	13	1 to 2	Sleep problems	0.24
8.2	Liu et al. (2021) ^b^	1,226	50%	13	2 to 3	Sleep problems	0.23
8.3	Liu et al. (2021) ^b^	1,226	50%	13	3 to 4	Sleep problems	0.33
9.1	Paloș et al. (2019) ^b^	142	24%	21	1 to 2	Grades	−0.14
10.1	Spagnoli et al. (2021) ^e^	191	44%	43	1 to 2	Perfectionistic concerns	0.22
11.1	Tóth-Király et al. (2021) ^b^	542	46%	23	1 to 2	Depression	0.11
11.2	Tóth-Király et al. (2021) ^b^	542	46%	23	2 to 3	Depression	0.12
11.3	Tóth-Király et al. (2021) ^b^	542	46%	23	3 to 4	Depression	0.13
12.1	Viotti et al. (2018) ^e^	155	-	-	1 to 2	Incivility	0.18
13.1	Viotti et al. (2019) ^e^	349	0%	49	1 to 2	Work ability	−0.16

S. E., study/effect number; Age, average age at first measurement; b, cross-lagged effect of the predictor on exhaustion/burnout reported in the article; ^e^ outcome, exhaustion; ^b^ outcome, burnout.

### Respondents

Sample sizes in the 13 studies varied between 142 and 2,235 (*M* = 856.5). Data were collected in eight different countries (China, Finland, Italy, Norway, Portugal, Romania, The Netherlands, and USA) and the populations included, for example, school children, firefighters, and general practitioners. Percentage of male participants varied between 0 and 90% (*M* = 49.1%) and mean age at the first measurement varied between 12.7 and 48.6 years (*M* = 36.4 years).

### Measures

The Maslach Burnout Inventory (MBI, Maslach et al., 1996) was the most commonly used instrument to measure burnout or exhaustion (in 7 of 13 studies). The predictors varied between the studies and were, consequently, measured with different instruments. However, the instruments appeared to have satisfactory homogeneity, with Cronbach’s alpha at the initial measurement varying between 0.66 and 0.92 (*M* = 0.81).

### Analyses

**Figure 1:**
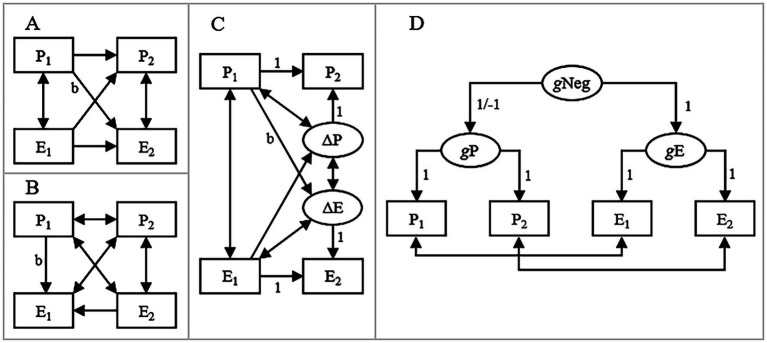
**(A)** Original cross-lagged panel model (CLPM), where initial predictor-value predicted subsequent burnout/exhaustion while adjusting for initial burnout/exhaustion and vice versa; **(B)** Reversed CLPM, where initial predictor-value predicted initial burnout/exhaustion while adjusting for subsequent burnout/exhaustion; **(C)** Latent change score model (LCSM), where initial predictor-value predicted subsequent latent change in burnout/exhaustion and vice versa; **(D)** A model of artifactualness without any direct effects between the predictor and burnout/exhaustion. P, predictor; E, burnout/exhaustion; 1 and 2 = initial and subsequent measurement, respectively; ΔP/ΔE, latent change in predictor and burnout/exhaustion respectively; *g*Neg, general negativity; *g*P/*g*E, general level of the predictor and burnout/exhaustion, respectively; 1/−1 = set to 1 or −1 if the predictor had a positive or a negative correlation with burnout/exhaustion, respectively; *b* = focal effect.

For each of the 23 effects in Table 1, we simulated data with the same sample size and six correlations between the predictor and burnout/exhaustion measured at two occasions. These correlations were reported in the reanalyzed studies and they are included in our descriptive dataset available at the Open Science Framework at https://osf.io/smy5n/. In each simulated dataset, the four variables (i.e., P_1_, P_2_, E_1_, and E_2_ in Figure 1) were drawn from a standard (*M* = 0, *SD* = 1) normal distribution. We did not include any missing values in the simulated data, meaning that procedures for handling missing data were not required. We used simulated data as the original data were not available to us. It is important to note that standardized regression effects are functions of correlations. The standardized effect of X_1_ on Y_2_ when adjusting for Y_1_ is given by Equation 1. (Cohen et al., 2003) and the effect of X_1_ on the Y_2_-Y_1_ difference score (all three variables standardized) is given by Equation 2 (Guilford, 1965). This means that if a simulated dataset has the same correlations between variables as an empirical dataset, regression effects estimated in the simulated dataset will be the same as if estimated in the empirical dataset. This is true even if the simulated dataset does not match the empirical dataset in some other regards, e.g., the distribution of the variables. Moreover, if the simulated dataset has the same sample size, the statistical significance of the regression effects will be the same as if estimated in the empirical dataset.

(1) $$\beta_{X1,Y2.Y1}=\frac{r_{X1,Y2}-r_{X1,Y1}r_{Y1,Y2}}{1-r_{X1,Y1}^{2}}$$

(2) $$\beta_{X1,Y2-Y1}=\frac{r_{X1,Y2}-r_{X1,Y1}}{\sqrt{2(1-r_{Y1,Y2})}}$$

**Figure 2:**
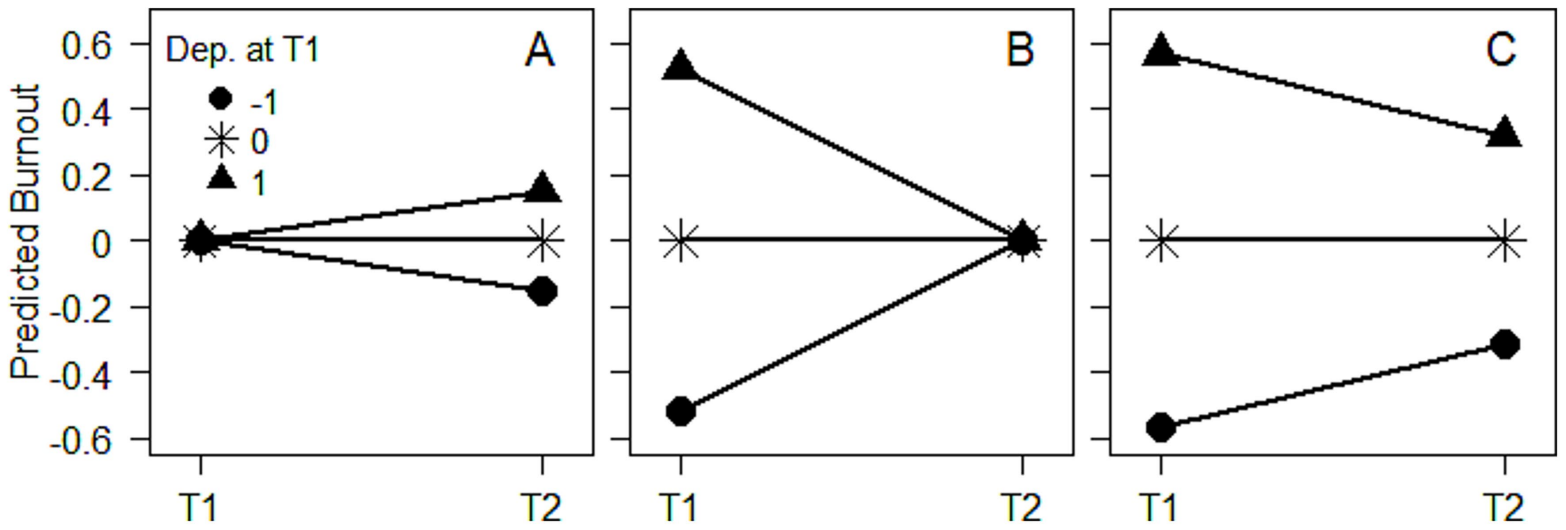
Predicted initial (T1) and subsequent (T2) burnout as functions of initial depression (Dep.) when: **(A)** Conditioning on average initial burnout; **(B)** Conditioning on average subsequent burnout; **(C)** Not conditioning on burnout. The conducted analyses, i.e., a cross-lagged panel model (CLPM), a reversed CLPM, and a latent change score model (LCSM), are illustrated in Figures 1A–C, respectively.

We fitted four complementary models to the simulated data: (1) A traditional CLPM where subsequent burnout/exhaustion was regressed on prior exhaustion/burnout and a prior score on the predictor and vice versa (Figure 1A); (2) A reversed CLPM where initial burnout/exhaustion was regressed on subsequent burnout/exhaustion and an initial score on the predictor (Figure 1B). This model was based on the logic that time-reversal should result in reversed signs of effects (Campbell and Kenny, 1999; Haufe et al., 2013). For example, if initial depression had an increasing effect on burnout, we should expect a negative effect of initial depression on concurrent burnout when adjusting for subsequent burnout. This negative effect would suggest that low initial depression had counteracted high initial burnout and allowed individuals to reach the same subsequent level of burnout as individuals who had a lower initial level of burnout but also a higher initial degree of depression; (3) A latent change score model (LCSM; McArdle, 2009; Ghisletta and McArdle, 2012; Kievit et al., 2018), where an initial score on the predictor predicted subsequent change in burnout/exhaustion and vice versa (Figure 1C). With a genuine increasing or decreasing effect of the predictor on burnout/exhaustion, this effect should be positive or negative, respectively; (4) A model of artifactualness, where initial and subsequent scores on the predictor and burnout/exhaustion were regressed on latent general predictor and burnout/exhaustion factors, respectively. These latent factors were, in turn, regressed on a second-order latent general negativity factor. Observed scores from the same occasion were allowed to correlate to account for presumed effects of various state factors, e.g., temporary mood (Figure 1D). A good fit of this model would indicate that data may have been generated by a model without any direct effects between the predictor and burnout/exhaustion, i.e., such effects may have been artifactual. In all analyses we used the lavaan default convergence criteria of 0.0001, meaning that model iterations stop when unscaled parameter values change less than 0.0001 (in absolute value)^1^.

Simulations and analyses were conducted with R 4.4.0 statistical software (R Core Team, 2025) using the MASS (Venables and Ripley, 2002), lavaan (Rosseel, 2012) and osfr (Wolen et al., 2020) packages. Data and the analytic script are available at the Open Science Framework at https://osf.io/smy5n/.

## Results

The size of the standardized focal effects (labeled *b* in Figures 1A–C) and fit of the model of artifactualness (Figure 1D) for each of the 23 reanalyzed effects are presented in Table 2. With some exceptions (discussed under Limitations below), the cross-lagged effect of initial predictor-score on subsequent burnout/exhaustion when adjusting for initial burnout/exhaustion tended to have a similar size and the same sign as the corresponding effect in the original study (compare effects in the “A” column in Table 2 with the “*b*” column in Table 1). For example, the positive effect of depression at T_1_ on burnout at T_2_ when adjusting for burnout at T_1_ was *b* = 0.11 in the study by Tóth-Király et al. (2021) (row 11.1 in Table 1) and *b* = 0.15 in our simulation (row 11.1 in Table 2). This effect suggested that among individuals with the same burnout at T_1_, those with higher depression at T_1_ had increased more in burnout between the measurements compared with individuals with lower depression at T_1_ (Figure 2A).

**Table 2:** Focal effects (labeled *b* in Figures 1A–C) and the fit of model D (Figure 1D) in data simulated to resemble data in the 13 reanalyzed studies (23 effects total, see Table 1 for references and characteristics).

S. E.	Focal effect [95% CI] in models A–C			Fit of Model D		
	A	B	C	*χ* ^2^	CFI	RMSEA [90% CI]
1.1	0.09 [0.02; 0.16]*	0.17 [0.10; 0.23]*	−0.05 [−0.12; 0.03]	0.6	1.00	0.00 [0.00; 0.00]
2.1	0.04 [0.00; 0.08]*	0.08 [0.04; 0.12]*	−0.03 [−0.08; 0.03]	1.4	1.00	0.00 [0.00; 0.00]
3.1	0.10 [−0.01; 0.20]	0.32 [0.23; 0.41]*	−0.14 [−0.26; −0.03]*	0.3	1.00	0.00 [0.00; 0.00]
3.2	−0.03 [−0.12; 0.07]	0.25 [0.17; 0.34]*	−0.19 [−0.31; −0.08]*	3.0	1.00	0.00 [0.00; 0.05]
3.3	0.09 [−0.01; 0.18]	0.18 [0.09; 0.27]*	−0.07 [−0.18; 0.05]	1.0	1.00	0.00 [0.00; 0.00]
4.1	0.08 [0.04; 0.12]*	0.33 [0.30; 0.36]*	−0.16 [−0.20; −0.12]*	0.4	1.00	0.00 [0.00; 0.00]
4.2	−0.01 [−0.04; 0.03]	−0.15 [−0.19; −0.12]*	0.10 [0.06; 0.14]*	0.7	1.00	0.00 [0.00; 0.00]
4.3	0.03 [0.00; 0.07]	0.13 [0.10; 0.17]*	−0.07 [−0.11; −0.03]*	0.9	1.00	0.00 [0.00; 0.00]
5.1	0.05 [0.01; 0.09]*	0.25 [0.22; 0.29]*	−0.13 [−0.18; −0.09]*	0.5	1.00	0.00 [0.00; 0.00]
5.2	0.06 [0.02; 0.11]*	0.26 [0.23; 0.30]*	−0.13 [−0.18; −0.09]*	0.9	1.00	0.00 [0.00; 0.00]
6.1	0.13 [0.09; 0.17]*	0.30 [0.27; 0.34]*	−0.11 [−0.15; −0.06]*	5.6	1.00	0.00 [0.00; 0.03]
7.1	0.13 [0.06; 0.19]*	0.31 [0.25; 0.37]*	−0.11 [−0.19; −0.04]*	0.7	1.00	0.00 [0.00; 0.00]
7.2	0.12 [0.05; 0.19]*	0.30 [0.25; 0.36]*	−0.12 [−0.19; −0.04]*	0.0	1.00	0.00 [0.00; 0.00]
8.1	0.16 [0.11; 0.22]*	0.29 [0.23; 0.34]*	−0.08 [−0.13; −0.03]*	10.3	0.99	0.02 [0.00; 0.05]
8.2	0.09 [0.04; 0.14]*	0.14 [0.09; 0.18]*	−0.03 [−0.09; 0.02]	50.6	0.96	0.08 [0.06; 0.10]
8.3	0.28 [0.23; 0.34]*	0.34 [0.28; 0.39]*	−0.04 [−0.09; 0.02]	1.2	1.00	0.00 [0.00; 0.00]
9.1	−0.14 [−0.26; −0.01]*	0.05 [−0.08; 0.18]	−0.13 [−0.30; 0.03]	3.3	1.00	0.00 [0.00; 0.07]
10.1	0.17 [0.04; 0.30]*	0.28 [0.16; 0.40]*	−0.07 [−0.21; 0.06]	0.9	1.00	0.00 [0.00; 0.00]
11.1	0.15 [0.05; 0.24]*	0.52 [0.45; 0.59]*	−0.25 [−0.33; −0.18]*	0.0	1.00	0.00 [0.00; 0.00]
11.2	0.22 [0.13; 0.30]*	0.44 [0.36; 0.51]*	−0.14 [−0.22; −0.06]*	0.2	1.00	0.00 [0.00; 0.00]
11.3	0.35 [0.26; 0.44]*	0.48 [0.40; 0.56]*	−0.09 [−0.16; −0.01]*	21.0	0.98	0.07 [0.04; 0.10]
12.1	0.16 [0.05; 0.27]*	0.03 [−0.09; 0.14]	0.09 [−0.06; 0.25]	1.3	1.00	0.00 [0.00; 0.00]
13.1	−0.21 [−0.30; −0.11]*	−0.33 [−0.42; −0.25]*	0.08 [−0.02; 0.18]	1.9	1.00	0.00 [0.00; 0.02]

CI, confidence interval; Statistically significant effects that align with effects and conclusions in the original studies are in italics while statistically significant effects that diverge from effects and conclusions in the original studies are in bold; * *p* < 0.05.

However, contrary to expectations in the case of genuine increasing or decreasing effects, the effect of initial predictor-score on initial burnout/exhaustion when adjusting for subsequent burnout/exhaustion tended to have the same sign as the effect on subsequent burnout/exhaustion when adjusting for initial burnout/exhaustion (compare effects in the “A” and “B” columns in Table 2). This suggested, for example, that high, not low, initial depression had counteracted high initial burnout and allowed individuals to reach the same subsequent burnout as individuals with lower initial depression (row 11.1 in Table 2 and Figure 2B).

Also contrary to expectations in the case of genuine increasing or decreasing effects, when significant, the effect of initial predictor-score on the subsequent latent change in burnout/exhaustion tended to have the opposite sign compared with the effect on subsequent burnout/exhaustion when adjusting for initial burnout/exhaustion (compare effects in the “A” and “C” columns in Table 2). This suggested, for example, that high, not low, initial depression was associated with subsequent decrease in burnout/exhaustion (row 11.1 in Table 2 and Figure 2C). A possible exception to this combination of contradictory effects was the effect of grades on burnout among students reported by Paloș et al. (2019) (row 9.1 in Table 2). Here, although not quite statistically significant, the effect of grades on subsequent latent change in burnout had the same sign (*b* = −0.13) as the cross-lagged effect of grades on subsequent burnout when adjusting for initial burnout (*b* = −0.14). Lastly, the model of artifactualness (Figure 1D) fitted the simulated data well in all of the 23 cases (e.g., CFI > 0.95, Table 2), indicating that data in the original studies may have been generated by a model without any direct effects between the predictor and burnout/exhaustion. Here (in Table 2), we report CFI and RMSEA because they are among the most popular and known fit indices. Additional fit indices, e.g., TLI and SRMR, are reported in the supplementary Table S1 available at the Open Science Framework at https://osf.io/smy5n/.

## Discussion

This study set out to evaluate concluded prospective effects on burnout and exhaustion in studies using the cross-lagged panel model. In data simulated to resemble data in the evaluated studies, we found contradictory increasing and decreasing effects of various predictors depending on the fitted model. These inconsistent findings suggested that the prospective effects may have been artifactual rather than genuine and, consequently, that conclusions by the authors of the original studies can be challenged. A possible exception was a concluded decreasing effect of good grades on burnout among students (Paloș et al., 2019). In many of the evaluated studies, authors drew implicit causal conclusions by expressing policy recommendations. For example, Viotti et al. (2019) suggested that their study “highlighted the importance of investing in promoting work ability in order to prevent job burnout (p. 898).” Based on the results in the present reanalyses, all such recommendations in the evaluated studies can be questioned.

As mentioned above, in a previous study we found, similarly as here, that various concluded prospective effects on work engagement did not appear to withstand closer scrutiny (Sorjonen et al., 2024b). Both of these studies with reanalyses are part of a more extensive set of studies where we have scrutinized and challenged conclusions of prospective effects between, for example, self-esteem and quality of social relations (Sorjonen et al., 2023a), self-esteem and work experiences (Sorjonen et al., 2023b), and social support and posttraumatic stress disorder (Sorjonen and Melin, 2023). A common theme in our challenging studies is that adjusted cross-lagged effects usually do not support causal conclusions any more than zero-order correlations do. This is a very important point for users of the cross-lagged panel model to keep in mind in order not to overinterpret findings. We recommend users of the cross-lagged panel model to scrutinize their findings by fitting, as we did here, complementary models to their data. If findings from the complementary models converge, conclusions are corroborated (although never finally proven). If, on the other hand and as in the present study, findings diverge, caution is advised and causal conclusions, explicit or implicit, should probably be avoided.

Researchers wishing to draw causal conclusions are recommended to carry out randomized controlled trials (RCT). If this is not possible, researchers are advised to interpret associations cautiously and preferably without causal language, i.e., in the form of policy recommendations. On this note, the present findings carry some practical relevance. For example, they warn decision makers not to listen too attentively to researchers making recommendations based on cross-lagged effects, e.g., in cross-lagged panel models. As shown here, cross-lagged effects do not prove causality. This means that following such recommendations have a high probability not to result in intended outcomes.

### Limitations

The point that adjusted cross-lagged effects may be artifactual has been made before (Campbell and Kenny, 1999; Glymour et al., 2005; Eriksson and Häggström, 2014; e.g., Castro-Schilo and Grimm, 2018; Sorjonen et al., 2019; Lucas, 2023). However, the output of studies using the cross-lagged panel model, often including uncritical causal conclusions, does not seem to subside. Therefore, reiteration of this point is warranted.

We recommend fitting complementary models to data, e.g., a time-reversed model where an initial score on the outcome is regressed on a subsequent score on the outcome in addition to an initial score on the predictor. However, the time-reversed model is just as susceptible to bias and artifactual findings as the traditional cross-lagged panel model. It is also possible, despite our arguments above, for the original and the time-reversed effects to have the same sign even in the presence of true causal effects (Sorjonen et al., 2024a). Moreover, positive and negative effects of a predictor on a latent change score of an outcome may be due to influence by unmodeled state factors rather than indicating true increasing and decreasing effects, respectively. With this in mind, we would not recommend claiming a decreasing effect of depression on burnout despite a negative effect being revealed by a latent change score model (Figure 2C). Consequently, with our recommendation to analyze complementary models we do not claim that they deliver infallible evidence of causality. However, it is our conviction that considering several fallible pieces of information make for better conclusions than considering just one fallible piece of information. We do not believe that it would be preferable to consider results only from the traditional cross-lagged panel model, which is susceptible to bias and artifactual findings, than from several complementary models. As an analogy, we do not believe that it would be tenable to argue that prosecutors should interview only one witness per case, instead of several, because human perception and memory is error-prone.

We used simulated rather than empirical data as the empirical data were not available to us. Somebody might consider this as a major limitation. However, we analyzed data with four complementary structural equation models (SEM, Figure 1) and SEM uses sample sizes and covariances or correlations (for standardized parameters) as input and estimates parameter values that minimize the difference between empirical covariances/correlations and covariances/correlations predicted by the defined model. Consequently, two datasets, empirical or simulated, with the same sample sizes and covariances/correlations between variables would yield very similar results. Therefore, the points made in the present study of questionable prospective effects on burnout/exhaustion cannot be explained away by our use of simulated data.

As mentioned above, SEM and path models, including the CLPM (Figure 1A), the reversed CLPM (Figure 1B), the LCSM (Figure 1C), and our model of artifactualness (Figure 1D), try to minimize differences between predicted and empirical covariances/correlations and fit indices reflect how good models are at minimizing this difference. However, correlations do not prove causality (Reichenbach, 1971). This means that statistically significant parameter values in SEM and path models, including directed regression effects, do not prove causality if the model has been fitted on observational (i.e., non-experimental) data, not even if the model has a good fit. For example, a negative effect of initial depression on subsequent latent change in burnout in a LCSM (effects 11.1–11.3 in Table 2) does not prove that depression has a true causal decreasing effect on burnout. Moreover, several alternative models may fit the same data well. This means that a well-fitting model may be the true data generating model but it does not have to be as other alternatives are possible. For example, a good fit of our model of artifactualness (Figure 1D) suggests that data may have been generated without any direct effects between predictors and burnout/exhaustion, but it does not prove that data must have been generated without such effects.

There were some discrepancies between our estimated cross-lagged effects on subsequent burnout/exhaustion when adjusting for initial burnout/exhaustion (column “A” in Table 2) and effects reported in the original studies (column “*b*” in Table 1). These discrepancies were presumably mainly due to adjustment for additional variables in the original studies rather than due to differences between the empirical and our simulated data. For example, the cross-lagged effect of initial work-to-family facilitation on subsequent exhaustion reported by Innstrand et al. (2008) (*b* = −0.24, *p* < 0.05, row 4.2 in Table 1) was estimated while adjusting for initial work-to-family conflict, family-to-work conflict, and family-to-work facilitation in addition to initial exhaustion. Our result (*b* = −0.01, *p* > 0.05, row 4.2 in Table 2) suggested that if Innstrand et al. (2008) had fitted a simpler model to their data, only adjusting for initial exhaustion, they would not have found a statistically significant cross-lagged effect of initial work-to-family facilitation on subsequent exhaustion. However, this does not mean that the effect presented by Innstrand et al. (2008) therefore, should be assumed to prove a true causal decreasing effect of work-to-family facilitation on exhaustion. “The effect was truly causal” does not follow deductively from “the effect was affected by adjustment for covariates.”

Moreover, the reversed effects in column B in Table 2 tend to be stronger than the “non-reversed” effects in column A. Adjustments for covariates tend to move regression effects closer to zero (although exceptions are possible) and we see no reason to assume that this effect should be stronger on the reversed compared with the non-reversed effects. Hence, there is no reason to assume that adjustments for covariates would “correct” the discrepant findings by moving the reversed effects to the other side of zero (i.e., changing the sign of the effect from positive to negative or vice versa) while, at the same time, allowing the non-reversed effects to remain on “the right” side of zero. The same reasoning can be applied on the discrepant effects on latent change scores in column C in Table 2. Consequently, the points made in the present study of questionable prospective effects on burnout/exhaustion cannot be explained away by us not adjusting for covariates.

Our selection of studies to reanalyze was not systematic. Instead, the set of selected studies could be characterized as a convenience sample. Consequently, it is possible, although hardly likely, that most studies using cross-lagged panel models to estimate prospective effects on burnout/exhaustion would, differently from the studies reanalyzed here, withstand closer scrutiny. However, even if that would be the case, the main methodological point of the present study, that effects in cross-lagged panel models do not prove causality, would still be valid.

## Conclusion

Many concluded prospective effects on burnout and exhaustion, based on analyses with cross-lagged panel models, appear to be artifactual. It is important for researchers to bear in mind that correlations, including effects in cross-lagged panel models, do not prove causality in order not to overinterpret findings. We recommend researchers to scrutinize findings from cross-lagged panel models by fitting complementary models to their data. If findings from complementary models converge, conclusions are corroborated. If, on the other hand, findings diverge, caution is advised and claims of causality, explicit or implicit, should probably be avoided.

## Data Availability

Publicly available datasets were analyzed in this study. This data can be found at: data and the analytic script are available at the Open Science Framework at https://osf.io/smy5n/.

## Footnotes

^1^ https://lavaan.ugent.be/history/dot6.html
